# Oxidative Stress in Patients with Type 1 Diabetes Mellitus: Is It Affected by a Single Bout of Prolonged Exercise?

**DOI:** 10.1371/journal.pone.0099062

**Published:** 2014-06-06

**Authors:** Maria Pia Francescato, Giuliana Stel, Mario Geat, Sabina Cauci

**Affiliations:** Department of Medical and Biological Sciences, University of Udine, Udine, Italy; University of Michigan Medical School, States of America

## Abstract

Presently, no clear-cut guidelines are available to suggest the more appropriate physical activity for patients with type 1 diabetes mellitus due to paucity of experimental data obtained under patients' usual life conditions. Accordingly, we explored the oxidative stress levels associated with a prolonged moderate intensity, but fatiguing, exercise performed under usual therapy in patients with type 1 diabetes mellitus and matched healthy controls. Eight patients (4 men, 4 women; 49±11 years; Body Mass Index 25.0±3.2 kg·m^−^^2^; HbA_1c_ 57±10 mmol·mol^−1^) and 14 controls (8 men, 6 women; 47±11 years; Body Mass Index 24.3±3.3 kg·m^−^^2^) performed a 3-h walk at 30% of their heart rate reserve. Venous blood samples were obtained before and at the end of the exercise for clinical chemistry analysis and antioxidant capacity. Capillary blood samples were taken at the start and thereafter every 30 min to determine lipid peroxidation. Patients showed higher oxidative stress values as compared to controls (95.9±9.7 vs. 74.1±12.2 mg·L^−1^ H_2_O_2_; p<0.001). In both groups, oxidative stress remained constant throughout the exercise (p = NS), while oxidative defence increased significantly at the end of exercise (p<0.02) from 1.16±0.13 to 1.19±0.10 mmol·L^−1^ Trolox in patients and from 1.09±0.21 to 1.22±0.14 mmol·L^−1^ Trolox in controls, without any significant difference between the two groups. Oxidative stress was positively correlated to HbA_1c_ (p<0.005) and negatively related with uric acid (p<0.005). In conclusion, we were the first to evaluate the oxidative stress in patients with type 1 diabetes exercising under their usual life conditions (i.e. usual therapy and diet). Specifically, we found that the oxidative stress was not exacerbated due to a single bout of prolonged moderate intensity aerobic exercise, a condition simulating several outdoor leisure time physical activities. Oxidative defence increased in both patients and controls, suggesting beneficial effects of prolonged aerobic fatiguing exercise.

## Introduction

Oxidative stress is a condition where the increased formation of free radicals overwhelms the available antioxidant capacity [1] and is involved in many human diseases, such as atherosclerosis, cardiovascular and neurological disease as well as in aging [1], [2]. Oxidative stress is also a widely accepted participant in the development and progression of diabetes mellitus (DM) and its complications [2], [3]. Indeed, disruption of the physiological free radicals homeostasis has been implicated in the impairment of beta-cell function [4], [5].

Regulated production of free radicals, however, participates in several physiological processes, being also beneficial [6]. In fact, free radicals act as a double-edged sword in modulating insulin signaling, being required for insulin to exert its physiological action, but also being implicated in the pathogenesis of insulin resistance [3]. There are multiple likely sources of reactive oxygen and nitrogen species (ROS/RNS), especially in diabetes, including glucose autoxidation, glycation of proteins, consumption of NADPH through the polyol pathway, and activation of protein kinase C [7], [8].

In patients with type 2 DM, an increased oxidative stress has been consistently shown [8] using different markers [7]. In contrast, in patients with type 1 DM both increases in oxidative stress [9]–[11], as well as unchanged levels [7], [12], [13] compared to healthy controls were reported.

Presently, there is evidence that regular physical activity can reduce the risk of several chronic diseases (e.g. cardiovascular disease, cancer, hypertension, obesity) and premature death, even in patients with diabetes [14]. However, for patients with type 1 DM, the available literature does not provide clear-cut guidance on the intensity, duration or type (aerobic/resistance) of physical activity that will offer the greatest benefits [15]. Notably, several patients perform only occasional outdoors leisure time physical activities (e.g., hiking, hill walking, biking, cross-country skiing), which tend to be prolonged in time, while being light to moderate in intensity. The effects of this kind of physical activity, however, are largely unexplored.

There is indirect evidence, on healthy subjects, that exercise may promote oxidative stress [1], [6]. The association of type 1 DM and exercise-induced free radicals production is still understudied [10], [16], although it is of great clinical and practical interest. In particular, data are lacking in patients exercising without modifying their usual therapy and/or as a result of a single bout of prolonged fatiguing exercise.

We studied the oxidative stress in a group of patients with type 1 DM at baseline and during a single bout of prolonged (3-h) moderate intensity aerobic exercise performed without modifying patient's usual insulin therapy or forcing a constant glycemia applying a clamp procedure, in comparison with a group of well-matched healthy controls. Lipid peroxidation (an indicator of oxidative stress) and the antioxidant defence were investigated by means of rapid point-of-care systems [2], [17].

## Materials and Methods

### Patients

Eight patients with type 1 DM (4 men and 4 women) and 14 age- and gender-matched healthy subjects (8 men and 6 women) were recruited. All subjects practiced some aerobic physical exercise regularly, but none was highly trained. All participants were informed of the nature, purpose, and possible risks involved in the study before giving their voluntary written consent to participate. The study was approved by the Ethical Committee of the University of Udine and conducted according to the Declaration of Helsinki.

Patients were accepted if they were not affected by other chronic diseases, had no evidence of diabetes complications contraindicating physical activity, and showed glycated hemoglobin (HbA_1c_) level ≤75 mmol·mol^−1^ (i.e. ≤9.0%) [18], [19]. All patients were on a basal-bolus insulin regimen; 5 patients used lispro insulin before meals, 3 used regular insulin. At bedtime, 7 patients used insulin glargine, while 1 used NPH insulin. Physical and clinical characteristics of the two study groups were summarized in Table 1.

**Table 1:** Demographic, clinical, and baseline biochemical parameters of the study subjects.

	Patients with type 1 DM	Healthy controls	p
Male/Female	4M-4F	8M-6F	
Age (y)	49.1±10.5	46.8±10.7	NS
Body mass (kg)	74.1±15.4	73.3±14.5	NS
Stature (m)	1.71±0.10	1.73±0.11	NS
Body Mass Index (kg·m^−2^)	25.0±3.2	24.3±3.3	NS
Duration of diabetes (y)	29.4±14.2	-	
Insulin dose (IU·kg^−1^ per day)	0.51±0.08	-	
HbA_1c_ (%)	7.4±1.0	5.7±0.5	<0.001
(mmol·mol^−1^)	57.0±10.4	38.3±5.0	<0.001
Creatinine (mg·dL^−1^)	0.86±0.10	0.99±0.19	NS
Uric acid (mg·dL^−1^)	2.7±0.7	4.3±1.4	<0.005
Total cholesterol (mg·dL^−1^)	181.6±32.5	189.2±34.3	NS
LDL-C (mg·dL^−1^)	116.9±24.3	118.6±29.9	NS
HDL-C (mg·dL^−1^)	53.1±12.4	55.6±12.8	NS
Triglycerides (mg·dL^−1^)	58.0±18.2	97.2±37.6	<0.05
ALT (U·L^−1^)^§^	16.5 (10–34)	13.5 (11–40)	NS
AST (U·L^−1^)^§^	30.0 (21–47)	25.0 (15–40)	NS
Homocysteine (mg·dL^−1^)^§^	9.1 (7.3–12.5)	11.0 (7.1–37.3)	NS
hs-CRP (mg·L^−1^)^§^	0.67 (0.39–1.75)	1.00 (0.06–3.21)	NS
Iron (µg·dL^−1^)	52.9±33.6	92.1±49.5	NS
Transferrin (mg·dL^−1^)	184.5±21.5	226.5±61.7	<0.05
Tf saturation (%)	44.6±8.4	33.1±16.2	NS
FORT (mg·L^−1^ H_2_O_2_)	95.9±9.7	74.1±12.2	<0.001
FORD (mmol·L^−1^ Trolox)	1.16±0.13	1.09±0.21	NS

Values are expressed as means ±SD. Parameters failing normal distribution (^§^) are shown as median value and range (in brackets) and the Mann-Whitney U test was used to test their statistically significant differences.

### Experimental protocol

All the volunteers were asked to refrain from physical activity, alcohol, and caffeine in the 24-h period preceding the experimental session. In addition, patients were advised to maintain their usual diet and insulin regimen and to carefully control their blood glucose levels according to the self-management procedures in order to avoid hypoglycemic events.

All volunteers attended the laboratory early in the morning. At 7:30 AM patients injected themselves with their usual insulin dose (0.08±0.04 IU·kg^−1^) subcutaneously in the abdomen wall and consumed their usual breakfast, which included ∼0.6 g·kg^−1^ of carbohydrates. Healthy control subjects had only a light breakfast at 7:30 AM, which included their usual average carbohydrate intake (∼0.7 g·kg^−1^). Thereafter, all participants rested in an armchair. About 45 min prior to the start of the exercise, an indwelling catheter was inserted into a forearm vein of the subject, patency of which was maintained by intermittent flushing with saline. Volunteers were then equipped with the belt of a heart rate monitoring system (Polar Electro, Kempele, Finland) to acquire heart rate (HR) values every 15 s throughout the exercise; HR data were subsequently downloaded and averaged over 15 min periods.

Volunteers performed a 3-h (from 10:00 AM to 1:00 PM) walk on a treadmill (Saturn, H-P Cosmos, Traunstein, Germany) maintaining HR constant by automatically adjusting speed and/or slope. To minimize dizziness due to the prolonged treadmill exercise, 5 min rest was allowed at the end of each hour of exercise. Work intensity was set to 30% of individual heart rate reserve (HR_target_ = HR_rest_+(HR_max_–HR_rest_)×0.3), where HR_max_ was estimated using the common equation 220 minus age (expressed in years). Higher work rates would likely increase the risk of hypoglycemia in patients and decrease compliance of volunteers to complete the task.

In patients, to counteract an excessive fall of glycemia, the algorithm of Francescato et al. [20], [21] was applied for each half-an-hour exercise to estimate the amount of required carbohydrates. Accordingly, patients were given 13±14 g (range 0–40 g) of fruit fudge about 30 minutes before the start of the exercise. Thereafter, additional amounts of fruit fudge (10±8 g; range 0–32 g) were administered to patients for each of the following 30 min exercise. Control subjects did not receive carbohydrates since there was no risk at all of hypoglycemia. All volunteers were allowed to drink water ad libitum.

Just before the start and at the end of the exercise, a venous blood sample (8.5 mL, and 5.5 mL, respectively) for clinical chemistry analysis and a capillary sample (50 µL) to determine the free oxygen radical defence (FORD tests; Calligari, Parma, IT) were drawn. Additionally, to determine lipid peroxidation (FORT tests; Calligari, Parma, IT), capillary blood withdrawals (20 µL) were taken just before the start of the exercise and, subsequently, at 30 min intervals. At the same times, glycemia was also tested in patients by using reactive strips on capillary blood (ContourLink, Bayer Healthcare Diabetes Division).

At the end of the trials, healthy control subjects were free to leave the laboratory. In contrast, to minimize the risk of late-onset hypoglycemic events [22], patients were kept under supervision of a physician trained in diabetes until the following morning. Patients received their usual insulin doses and meals for lunch and dinner (at 1:30 PM and 7:30 PM, respectively) and were allowed to sit quietly in an armchair (or lay in bed) while their glycemia was tested by means of reactive strips at least hourly. All the glycemic levels were recorded on appropriate forms, the analysis of which confirmed us that late-onset hypoglycemia after the trials was actually avoided in all patients.

### Analyses

Venous blood was collected in a tube with gel and clot activator (Vacutainer SST II advance #368965), a tube containing a glycolysis inhibitor (4 mg kalium oxalate +5 mg sodium fluoride; Vacutainer #368920), and a tube containing EDTA (Vacutainer #368856). Immediately after collection, tubes were gently mixed and the hospital laboratory processed the samples within 1 hour from collection.

Plasma glucose concentration was determined by applying a hexokinase based methodology (Olympus Diagnostic Systems AU2700; Olympus America Inc., Miami, FL, USA); our determined coefficient of analytical variation was <2% in the range 3.27 to 11.67 mmol·L^−1^.

Insulin concentrations were determined by the DxI800 Automated Immunoassay system (Beckman Coulter, Fullerton, CA, USA) (coefficient of analytical variation was <6% in the range 11.08 to 49.13 IU·L^−1^), which measures all the relevant insulin analogs with a cross-reactivity of about 80% [23].

HbA_1c_ was determined using the G8 HPLC Analyzer (Tosoh Biosience Inc, S. Francisco, CA, USA). Our determined coefficient of variation was 1.06% at an average concentration of 33 mmol·mol^−1^ (5.2%). Samples from two control subjects were unavailable for HbA_1c_ analysis.

Concentrations of uric acid, homocysteine, triglycerides, total cholesterol, HDL cholesterol, ALT, AST, and hs-CRP were measured on Olympus AU5400 analyzer (Olympus America Inc., Miami, FL, USA) by original reagents. LDL cholesterol was calculated from total cholesterol, triglycerides, and HDL according to the Friedewald equation.

Reactive oxygen species, in the form of lipid peroxides, were determined using a colorimetric assay called FORT test (Callegari, Parma, Italy) [2], [24]–[27]. Results were expressed as FORT units, whereby 1 FORT unit corresponded to 0.26 mg·L^−1^ H_2_O_2_. Our determined coefficient of analytical variation was <3.6%. Blood antioxidant capacity was determined by the colorimetric FORD test (Callegari, Parma, Italy) [2], [24]. The absorbance values of the samples were compared with a standard curve obtained using Trolox, a derivative of vitamin E commonly used as an antioxidant. Coefficient of analytical variation was <5.0%.

### Statistical Analysis

All variables were tested for normal distribution. Differences between patients and controls were examined using the Student t test or the Mann-Whitney U test as appropriate.

For multiple comparisons, analysis of variance for repeated measures (MANOVA) was applied with time as within-subjects factor and study group as between-subjects factor, followed by specific contrasts when appropriate. The two-tailed Spearman correlation coefficient for nonparametric variables was used to evaluate associations between the plasma concentrations of FORT with the study parameters. Two-tailed p<0.05 was considered significant. Systat version 11 software (Systat Software GmbH, Erkrath, Germany) was used. Values were expressed as means±SD or median and range, as appropriate.

## Results

Demographic characteristics and baseline biochemical parameters of the 8 patients and 14 controls were described in Table 1. There were no significant differences between patients and controls for most variables. Eight subjects were overweight but none was obese (Body Mass Index<30 kg·m^−2^). Values of serum biomarkers did not differ significantly between the two groups, with the exception of uric acid, triglycerides, and transferrin, all of which were lower in patients.

Actual exercise intensity, expressed as percentage of individual's heart rate reserve, was not significantly different between patients and controls (p = NS), amounting on average to 28.8±2.3% in patients and 31.4±4.1% in controls, corresponding to a grand-average value of 101±4 bpm. Progression speed was not significantly different between the two groups (group effect, F = 0.43, p = NS), showing a slight decrease across exercise duration from 5.3±0.6 km·h^−1^ to 5.0±0.7 km·h^−1^ (time effect, F = 1.980, p<0.05). Total traveled distance was 15.7±1.5 km, with no difference between the two groups.

**Figure 1:**
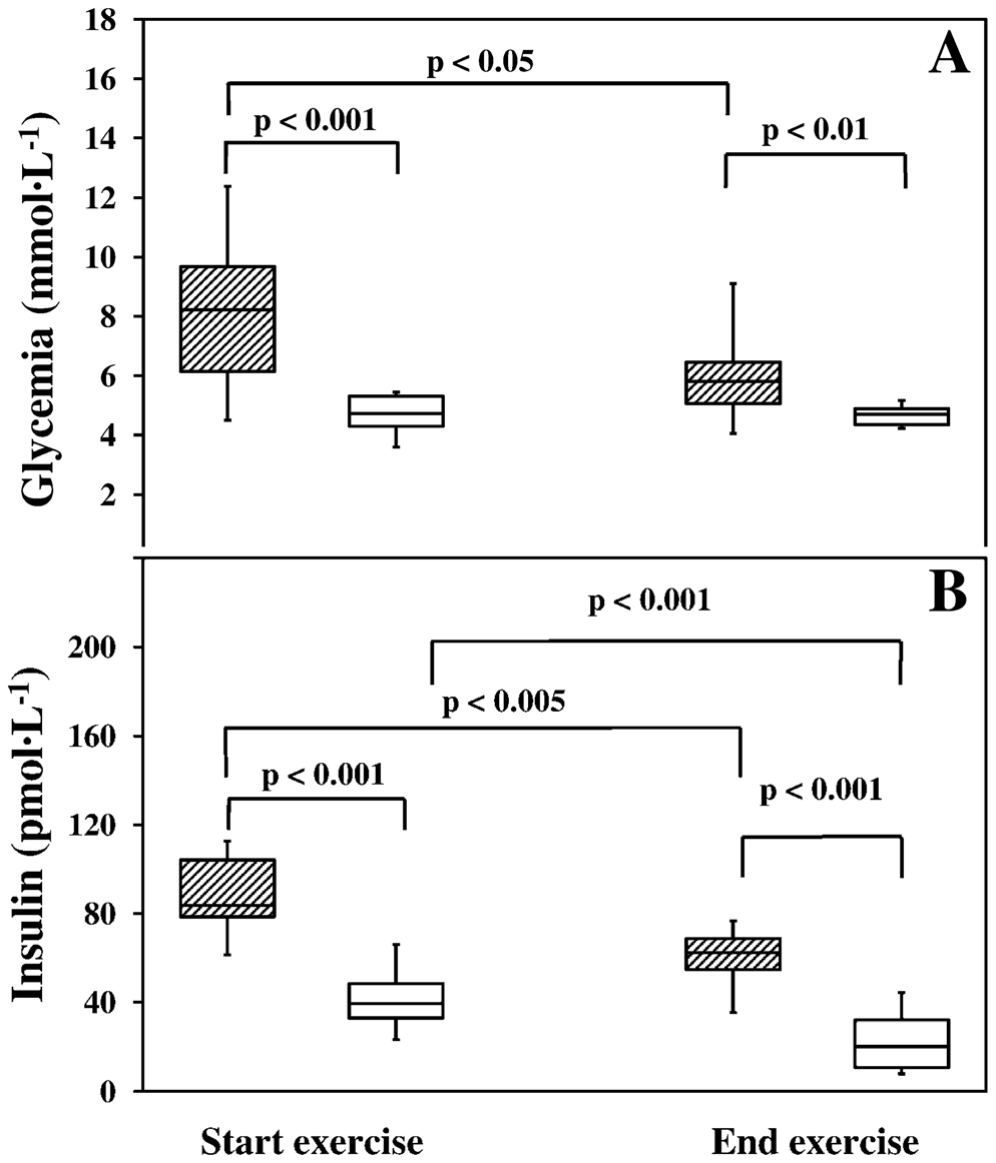
Glycemia and insulin concentration before and at the end of the 3-h exercises. Panel A. Glycemia (mmol·L^−1^) was significantly higher in patients both before and at the end of the exercise (p<0.01) and decreased significantly in patients with type 1 DM at the end of the trial (p<0.05). Panel B. Insulin concentration (pmol·L^−1^) was significantly higher in patients both before and at the end of the exercise (p<0.001) and decreased significantly in both groups at the end of the trial (p<0.005). In both panels: shaded boxes = patients, open boxes = healthy control subjects.

Both at the start and the end of exercise, glycemia was significantly higher in patients than in controls (Figure 1, panel A; group effect, F = 19.04, p<0.001). Glycemia followed a different behavior in the two groups (group × time effect, F = 11.22, p<0.005); in controls glycemia did not change significantly throughout the exercises (time effect, F = 0.01, p = NS) amounting on average to 4.6±0.5 mmol·L^−1^, whereas in patients glycemia fell significantly from 8.0±2.7 mmol·L^−1^ at the start to 5.9±1.6 mmol·L^−1^ at the end of the exercise (time effect, F = 7.21, p<0.05).

Figure 1, panel B, illustrates the significantly higher insulin concentrations of patients as compared to controls before and at the end of the exercise (group effect, F = 81.87, p<0.001). In both groups, insulin concentration decreased significantly at the end of the exercise (time effect, F = 40.88, p<0.001).

**Figure 2:**
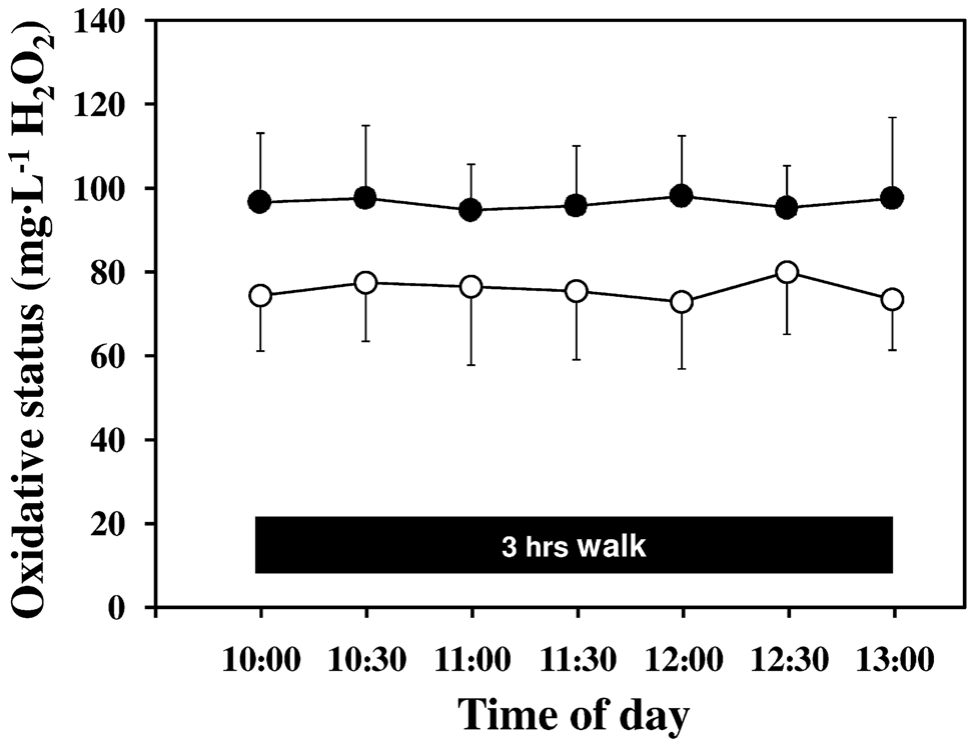
Oxidative stress values throughout the 3-h exercises. Oxidative stress was significantly higher in patients (p<0.005), but remained essentially unchanged in both groups across the exercise (p = NS). Full dots = patients with type 1 DM, open dots = healthy control subjects.

Figure 2 shows that oxidative stress (FORT) values remained constant in both groups of volunteers throughout the 3-h exercise (time effect, F = 0.21, p = NS). At each time point, oxidative stress was significantly higher in patients than in controls (group effect, F = 14.67, p<0.001). On the opposite, in both groups, total antioxidant capacity (FORD) values increased significantly from baseline to the end of the exercise (1.16±0.13 vs. 1.19±0.10 mmol·L^−1^ Trolox in patients and 1.09±0.21 vs. 1.22±0.14 mmol·L^−1^ Trolox in controls; time effect, F = 6.73, p<0.02) without any significant difference between the two groups (group effect, F = 0.11, p = NS).

**Figure 3:**
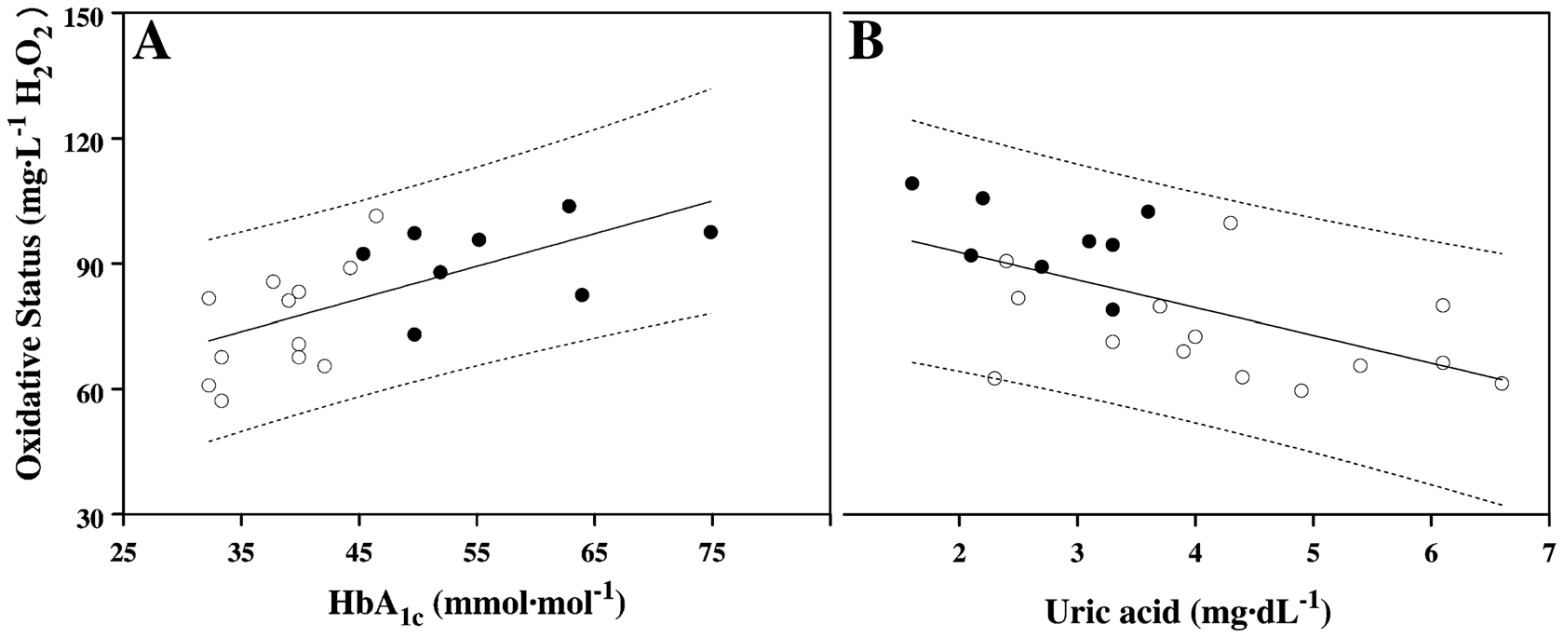
Relationship between oxidative stress and HbA_1c_ or uric acid. Panel A. Relationship between baseline HbA_1c_ values (mmol·mol^−1^) and oxidative stress (mg·L^−1^ H_2_O_2_). The relationship is statistically significant (n = 20, R = 0.649, p<0.005). Panel B. Relationship between baseline uric acid values (mg·dL^−1^) and oxidative stress (mg·L^−1^ H_2_O_2_). The relationship is statistically significant (n = 22, R = 0.621, p<0.005). In both panels: full dots = patients with type 1 DM, open dots = healthy control subjects, dotted lines = 95% confidence limits.

Average oxidative stress values were positively related to the HbA_1c_ values (Figure 3, panel A; n = 20, R = 0.649, p<0.005), and a negative relation with serum concentration of uric acid was found (Figure 3, panel B; n = 22, R = 0.621, p<0.005). No significant correlation was found between oxidative stress and the triglycerides or the transferrin levels.

## Discussion

The present study first showed that a single bout of prolonged moderate intensity aerobic exercise did not increase the lipid peroxidation levels (FORT test, as marker of oxidative stress), in both patients with type 1 DM and healthy subjects, despite higher peroxidation levels were observed throughout in patients. In contrast, an increase in the anti-oxidant defence (evaluated by the FORD assay) was observed at the end of the exercise in both patients and controls.

Physical exercise is strongly recommended in DM patients because of its numerous health benefits [15], but it might also cause oxidative stress [8], resulting in a potentially harmful condition [1]. Indeed, accumulating evidence suggests that oxidative cell injury caused by free radicals contributes to the development of DM complications [3], [5], thus patients should avoid situations that might increase oxidative stress. Our results suggest that the 3-h exercise performed in the present study, which simulate several outdoor leisure time physical activities, although fatiguing, were not of sufficient intensity to provoke a large enough increase in free radical production to overwhelm the antioxidant defences [1], [6]. Conversely, other authors found that an exercise at higher intensity and/or at exhaustion increased the oxidative stress levels in type 1 DM patients [10], [16].

The observed constancy of the oxidative stress during the 3-h exercise provided new information on the effects of a single bout of aerobic exercise both in the general population and type 1 DM patients, which are considered more prone to oxidative stress effects [16]. Indeed, contractile activity is known to cause an increase in ROS generation in muscle, but the factors influencing the magnitude of this response include also the nature and the duration of the contractile activity [28]. Thus, we can speculate that our exercise condition was not able to induce a significant increase in free radicals production. On the other hand, the investigated physical activity resulted in a significant increase in the serum antioxidant capacity, suggesting that the pathways that generate free radicals and those stimulating the antioxidant defence are in some way unrelated, as recently observed by other authors [24]. Indeed, during moderate exercise, ROS act also as signals resulting in an upregulation of powerful antioxidant enzymes such as superoxide dismutase, glutathione peroxidase and catalase [29], [30]. In general, ROS/RNS generated during muscle contraction appear to have a physiological role in the adaptation to exercise, leading to the view that moderate exercise can be considered also an antioxidant with beneficial effects [30], [31]. Our results induce to postulate that the same conclusion can apply also to patients with type 1 DM. Main purpose of our study was the investigation of lipid peroxidation; accordingly, only a general test, i.e. the FORD assay, was used to obtain overall information about the anti-oxidant activity. In the future, to depict in greater detail the effects of a prolonged exercise, it will undoubtedly be of great interest to investigate the activity of specific enzymes.

It should be pointed out here that patients with type 1 DM often require some extra carbohydrates before/during the effort to prevent an excessive fall of glycemia [21], even when they reduce the dose of injected insulin in anticipation of exercise [32]. This extra amount of carbohydrates might be considered a caloric load that will be oxidized in mitochondria, resulting in a potential higher production of free radicals and thus constituting a confounding variable in the experimental setup of the present work. Nevertheless, an experimentation similar to the present one [33] showed that the whole-body carbohydrates oxidation rate was not significantly different between patients with type 1 DM receiving appropriate amounts of fruit fudge (to avoid an excessive fall of glycemia) and the control group, who was not given carbohydrates during the exercise. In addition, it was reported by others that increased extracellular glucose availability does not necessarily translate into increased intracellular glucose oxidation during exercise [34], [35]. Furthermore, Chokkalingam et al. [36] observed that only patients exercising under high insulin infusion rates showed a slightly increased whole-body carbohydrates oxidation rate. Accordingly, we believe that the administration of fruit fudge to our patients during the 3-h walks had only a negligible effect on their oxidative stress levels.

The positive relationship we observed including all study subjects (patients and healthy controls), between oxidative stress and glycated hemoglobin (HbA_1c_) levels was unexpected. Our finding, however, is in line with the loose relationship (p = 0.02) between urinary 8-hydroxydeoxyguanosine concentration and HbA_1c_ in patients with type 1 DM reported by others [37]. Indeed, moderate elevations of glucose can potentially affect the oxidative stress [38]. Nevertheless, only rather prolonged periods of improved glycemic control induced a reduction in the oxidative stress in patients with diabetes [18], [19], while no similar observations have ever been reported for acute changes of glycemia occurring within a few hours. Thus, the constancy of oxidative stress levels observed in our patients at the end of the 3-h walks, despite a clear fall of their glycemia, is not surprising.

An unexpected finding was also the negative relationship, including all study subjects, between oxidative stress and serum uric acid levels. Indeed, uric acid is a powerful antioxidant and scavenger of singlet oxygen and radicals [39], [40]. The lower uric acid levels in patients in comparison to controls were consistent with few previous findings where a significantly reduced level of plasma uric acid amongst subjects with type 1 DM was reported [9], [13]. It remains to be clarified whether the low uric acid concentration observed in patients is a consequence or a cause of the high oxidative stress levels observed in these subjects. Although future research is warranted to better evaluate this relationship on a larger number of patients, clinicians should be suggested to carefully monitor uric acid, due to its association with the oxidative stress, in particular in patients with type 1 DM.

In our work, lipid peroxidation was used as marker of ROS [17] because of their interesting association with atherosclerosis and cardiovascular disease [41], but, on the other hand, it might represent a limitation of the present study. In fact, the observed levels of oxidative stress might depend also upon the kind of investigated oxidative stress marker [7], because reactive oxygen species are a vast group of agents, which are usually short lived and often difficult to measure. We choose the FORT and FORD tests because of their simplicity and rapidity of the assays, requiring only a small drop of capillary blood, thus allowing repeated and minimally invasive measuring. According to Kamhieh-Milz et al. [24], we are confident that the FORT and the FORD tests provide actually reliable results. Among others, the FORT test was also used to investigate the association between oxidative stress and C-reactive protein levels among healthy adults [25], to evaluate the effects of an innovative treatment in cancer-related anorexia/cachexia [27] and to investigate oxidative stress in different populations with type 2 diabetes [2], [26]. It appears thus that measurement of oxidation markers by means of point-of-care methods may be helpful to assess oxidant activity and to monitor the effectiveness of the antioxidant system in several different physiological conditions including exercise.

The rather small number of investigated patients might represent a further limitation. This was the consequence of the challenging protocol, especially for patients who had to be monitored for several hours following the exercise for the high risk of severe late-onset hypoglycemia. To partially compensate for this small number, we recruited a number of controls almost twice the patients. Furthermore, the small number of examined subjects did not allow us to investigate eventual gender differences [42].

## Conclusions

Results of the present study showed that the 3-h walking exercise did not worse the oxidative stress levels, suggesting that a single bout of prolonged moderate intensity aerobic exercise can be performed as leisure time outdoors activity also by patients with type 1 diabetes, despite they show a baseline oxidative stress higher than healthy controls. In the mean time, oxidative defence increased in both patients and controls, suggesting beneficial effects also of prolonged fatiguing but not exhausting exercise. Finally, the role of uric acid and chronically elevated glycemia in patients with type 1 DM in relation to oxidative stress should be investigated in greater detail.
